# Universality of the emergent scaling in finite random binary percolation networks

**DOI:** 10.1371/journal.pone.0172298

**Published:** 2017-02-16

**Authors:** Chongpu Zhai, Dorian Hanaor, Yixiang Gan

**Affiliations:** 1 The School of Civil Engineering, The University of Sydney, Sydney, New South Wales, Australia; 2 Institute for Materials Science and Technology, Technische Universität Berlin, Berlin, Germany; Consejo Nacional de Investigaciones Cientificas y Tecnicas, ARGENTINA

## Abstract

In this paper we apply lattice models of finite binary percolation networks to examine the effects of network configuration on macroscopic network responses. We consider both square and rectangular lattice structures in which bonds between nodes are randomly assigned to be either resistors or capacitors. Results show that for given network geometries, the overall normalised frequency-dependent electrical conductivities for different capacitor proportions are found to converge at a characteristic frequency. Networks with sufficiently large size tend to share the same convergence point uninfluenced by the boundary and electrode conditions, can be then regarded as homogeneous media. For these networks, the span of the emergent scaling region is found to be primarily determined by the smaller network dimension (width or length). This study identifies the applicability of power-law scaling in random two phase systems of different topological configurations. This understanding has implications in the design and testing of disordered systems in diverse applications.

## Introduction

The bulk behavior of complex systems comprising disordered multi-phase components is of importance in diverse applications including supercapacitors and batteries [1–4], dielectric material characterization [5–10], the mechanics of structures [11–13], fracturing process [14], thermal analysis [15] and soil probing [16]. In such systems, various parameters govern the electrical, thermal, chemical, and/or mechanical properties of components of a system across multiple scales from molecular up to macroscopic length-scale. Experimental and computational research efforts are increasingly conducted in order to gain insights into the manner in which these properties combine across scales to determine overall system performance.

**Fig 1:**
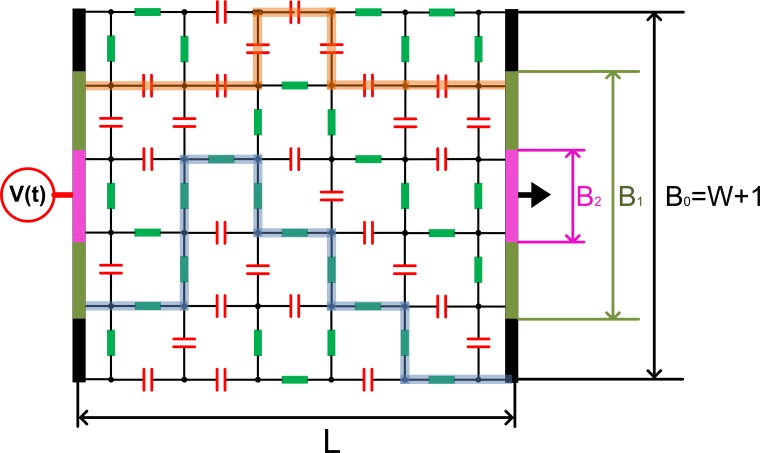
A lattice network containing *W* × *L =* 5 × 5 randomly distributed resistors and capacitors. The network width *W* and length *L* indicate the numbers of horizontal and vertical elements in a single chain, respectively. The values of *B*_0_ = 2, *B*_1_ = 4, and *B*_2_ = 6 present the number of nodes connected directly to the electrodes. Percolation paths formed by resistors and capacitors are shown in thick blue and orange lines, corresponding respectively to the dominating modes at low and high frequencies, for the *B*_*0*_ configuration.

In particular, the AC conductivity of systems that can be schematically represented as mixtures of electrical components has been the subject of numerous investigations that have shown power-law scaling with frequency arising through different relaxation mechanisms [11, 17–19]. Above a critical frequency this scaling of AC conductivity is described by Jonscher’s power law[20] and has been experimentally observed across diverse conductor-dielectric composites and porous materials [5, 7, 18, 21, 22] with this scaling being termed the “Universal Dielectric Response” [17, 18, 21, 23]. This emergent property does not arise directly from any particular physical or chemical properties of the involved components, but rather is a consequence of the way components combine [11, 19, 21, 24, 25]. Such dielectric mixtures have been effectively approximated as a random network of resistors and capacitors [18, 19, 24] with representative conductors exhibiting a constant conductance 1/*R* and dielectric components exhibiting a variable complex admittance *iωC*, which is directily proportional to an angular frequency *ω*, as illustrated in Fig 1. Useful asymptotic formula for the emergent network admittance including both the effects of component proportions and the network size can be obtained based on the spectral method [21, 26] and the averaging approach [11, 21, 27]. However, establishing a more rigorous estimation necessitates numerical analysis.

From previous numerical studies [11, 17–19, 21, 23, 25, 28], the typical obtained conductivity-frequency spectrum of a square lattice resistor-capacitor (RC) network can be divided into three regions of angular frequency, *ω*, governed by the proportion of capacitors, *p*_*c*_, and network size, *N* (a) an emergent region for intermediate frequencies; (b) two percolation regions; (c) transitions between the two above-mentioned [21]. Symmetry is found between the overall responses at high and low frequencies, which can be correlated to percolation behavior [26, 29, 30]. For low and high frequencies, current tends to percolate predominantly through resistors and capacitors respectively, as these will exhibit relatively lower impedance, as presented in Fig 1. For intermediate frequencies, where the values of admittance for the resistors and the capacitors are close, we observe the power-law emergent behavior whereby conductivity is proportional to *ω*^*α*^, with *α ≈ p* [11, 21, 27].

Motivated by the applicability of these networks for representing real-world disordered systems, in this paper we reexamine the universality of the emergent power-law scaling observed in previous work by further considering the significance of the network aspect ratio and boundary conditions on the convergence point and the span of the emergent region, the two key network characteristics of universal scaling behavior.

## Methods

In this paper, we extend the square lattice RC networks to rectangular ones with *N* = *W* × *L* elements distributed between two bus-bars, one of which is grounded and the other raised to a potential, |*V*_0_|*e*^*iωt*^. A system of complex number linear equations is set up by applying Kirchhoff’s current law (for complex currents) on each individual node of the RC network. For the node *k*, we get

$$I_{k}\left(t\right)=\Sigma_{j}^{n}\left|I_{j,k}\right|e^{i\left(\omega t+\varphi_{j,k}\right)}=\Sigma_{j}^{n}\left(\left|V_{j,k}\right|e^{i\left(\omega t+\varphi_{j,k}\right)}-\left|V_{k}\right|e^{i\left(\omega t+\varphi_{k}\right)}\right)/Z_{j,k}=0,$$ (1)

where *I*_*k*_ is the sum of the currents (negative or possitive) flowing towards the node *k*, from connected components. The impedance of a component connected to node *k*, *Z*_*j*, *k*_, is randomized to be either *R* or 1/*iωC*. The voltage potential of the connected node *j*, $\left|V_{j,k}\right|e^{i\left(\omega t+\varphi_{j,k}\right)}$, with respect to that of the node *k*, is represented as complex-valued function of time, *t*, with *φ*_*j*, *k*_ being the relative phase. The value of *n* is determined by the location in the network, equaling the number of connected components. More specifically, *n* = 4 for ones located away from the boundaries, *n* = 2 for the lattice corners, *n* = 3 for the nodes on the boundaries excluding corners. The two electrodes are also regarded as nodes with *n* = *B*. The electrode dimension, *B*, is defined as the number of nodes connected directly to the electrodes, e.g., *B* = *W* + 1 when all the elements along the boundary side are connected to the electrode. Each single node is represented by a corresponding linear algebraic equation, resulting in (*W* + 1) × (*L* + 1) equations for all the nodes. Two additional equations can be obtained from the electrodes. By solving these equations, the potential of each node and the current going through each bonds in the network can be calculated. Thus, for a given applied potential difference between electrodes, |*V*_0_|*e*^*iωt*^, the macroscopic admittance can be given by $Y=\left|I_{0}\right|e^{i\left(\omega t+\varphi_{I}\right)}/\left|V_{0}\right|e^{i\omega t}$, where $\left|I_{0}\right|e^{i\left(\omega t+\varphi_{I}\right)}$ is the obtained overall current flowing into the ground. Additionally, Frank-Lobb techniques can be employed to reduce the network size, thus improving the computational efficiency [31].

**Fig 2:**
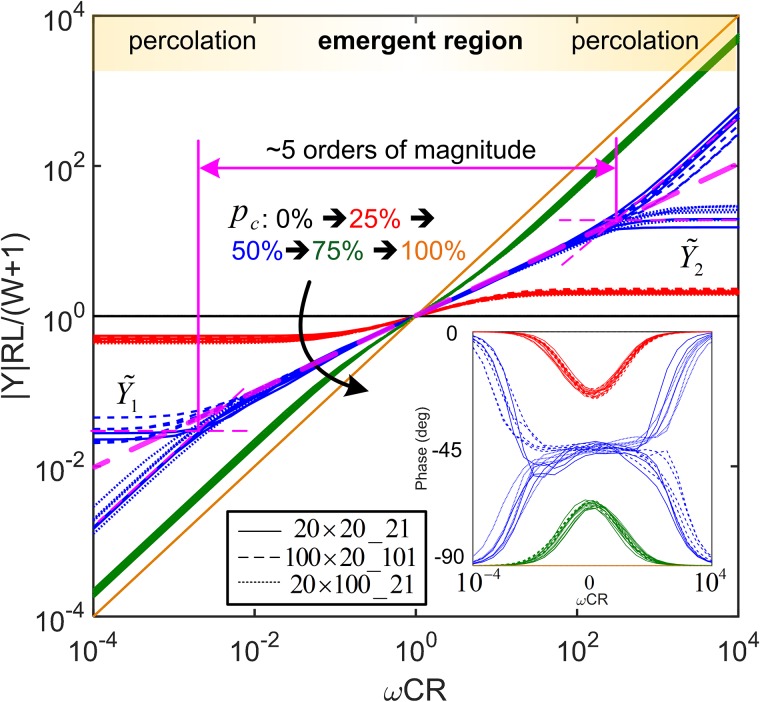
Normalised admittance module as a function of frequency. Numerical results obtained from three groups of differently configured networks (denoted as *W* × *L_B*) are presented, with capacitor proportions, *p*_*c*_, varying from 0% (corresponding curves are shown in black) to, 25% (red), 50% (blue), 75% (green), and to 100% (brown). The phase responses are depicted in the inset. For each network configuration with a given capacitor proportion, five simulations have been realized.

Here, the network aspect ratio and the electrode dimension are considered as variables, in order to investigate the influence of network configuration and boundary conditions on macroscopic responses. The network size is determined by its width, *W*, and length, *L*, which represent the numbers of components in a single chain along the horizontal and vertical directions, respectively. In the network circuit, two electrodes of identical dimension are connected to elements located symmetrically in the center of the two vertical boundaries. The frequency-dependent macroscopic responses obtained from different configurations, in terms of network length, *L*, and width, *W*, are normalized through

$$\tilde{Y}=\frac{\left|Y\right|RL}{W+1},\;\;\tilde{\omega }=\omega RC,$$ (2)

where *R* and *C* are the resistance and capacitance values of resistors and capacitors in the considered network, respectively. This normalization process is applied in order to include the significance of all the elements in the overall network behavior, represented by the equivalent admittance, by considering rules for simple series and parallel combinations of components. The span of the emergent region, *S*, is defined as the horizontal distance between the intersections of the power-law function, $y=\omega^{p_{c}}$, with top and bottom percolation admittances, $\tilde{Y_{1}}$ and $\tilde{Y_{2}}$ (averaged from multiple simulations), for low and high frequencies, respectively, as shown in Fig 2.

The network behaviors shown in Fig 2 in the frequency domain are primarily governed by percolation effects [32], which are closely linked to the frequency-dependent conductivity of each single bond in the network. In the studied rectangular RC networks with two types of bonds (i.e., resistor and capacitor, the admittance ratio of capacitor elements with respect to resistors is *iωRC*) have been considered to describe the responses of random binary networks. The observed universal scaling behavior in Fig 2 can be also found in other networks containing two types of elements, indexed by *a* and *b*, exhibiting differences which can be described in the form of *S*_*a*_ = ω*S_*b*_, e.g., mechanical stiffness, thermal conductivity, and chemical reaction rate, etc. [11, 14, 25].

## Results and analysis

The AC electrical responses of three groups of different sized networks (expressed in the form of *W* × *L_B*, representing network width×length_electrode dimension: 20 × 20_21, 100 × 20_101, and 20 × 100_21) with varying *p*_*c*_ are plotted in Fig 2. The three types of regions observed in the conductance spectroscopy of square networks can be also observed here in rectangular ones. For a given network geometry, the obtained admittance spectroscopies for various *p*_*c*_, are found to intersect at a convergence point with the characteristic frequency of *ω* = 1/*RC* where resistors and capacitors contribute equally to the overall conduction. This point also appears to be the center of the emergent region. The normalized characteristic admittance (the values of $\tilde{Y}$ at the characteristic frequency) at the convergence point is close to 1. This indicates that the network at the characteristic frequency perform effectively as a mono-element network, which has a phase angle reaching the extremum value, as is shown in Fig 2. It is further evidenced that the normalized emergent regions of the three groups of networks coincide with each other presenting universal features. The normalized admittance in this common emergent region appears to be uninfluenced by the network aspect ratio (length/width) or the electrode dimension. However, differences can be found at percolation regions along with the corresponding transition regions. Variation of the width or length can potentially change the percolation thresholds which will determine the responses, following a resistive-percolated (plateaued) or capacitive-percolated (upwards or downwards) trend, corresponding to the low and high frequency ranges, respectively.

**Fig 3:**
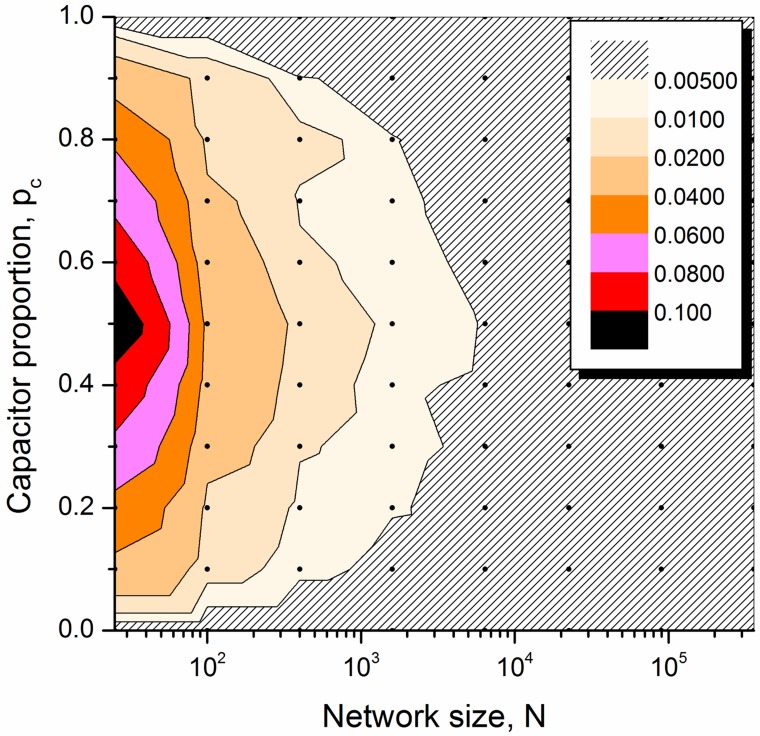
Mapping of the standard deviation of normalised characteristic admittance values. For varying capacitor proportions from 0.1 to 1.0, different-sized square networks (from 5 × 5 to 600 × 600) were considered. For a given network size and capacitor proportion, ten RC networks were generated and used in the simulations to obtain the averaged normalised characteristic admittance, represented by the black dot. The STD of these points are used for mapping with the colour indicating the STD values, as detailed in the legend.

Statistical analysis of the normalised characteristic admittance for different-sized square networks (from 5 × 5 to 600 × 600) with *B* = *W* + 1 was conducted and the standard deviation (STD) of the normalized characteristic admittance is presented in Fig 3. It is found that all values of normalized characteristic admittance obtained with various *p*_*c*_ (averaging over ten simulations) are in the range of (0.95, 1.05) for networks with more than 10 × 10 elements. The variance tends to diminish as the network size increases. This can be explained by considering boundary effects that relatively smaller networks have higher percentage of boundary elements (connected to four other elements rather than six in the bulk region). Responses of larger networks perform with little influence from the boundary. For a given sized square network a larger variation is found for cases of *p* = 1/2, as such conditions lead to an equal likelihood of resistive-percolated and capacitive-percolated network responses at low and high frequencies. Consequently, there are four possible qualitatively different types of response for any realization of the system.[21] Different available responses potentially introduce dispersion and uncertainty of the network behavior in both percolation and emergent regions, as can be seen in Fig 3.

**Fig 4:**
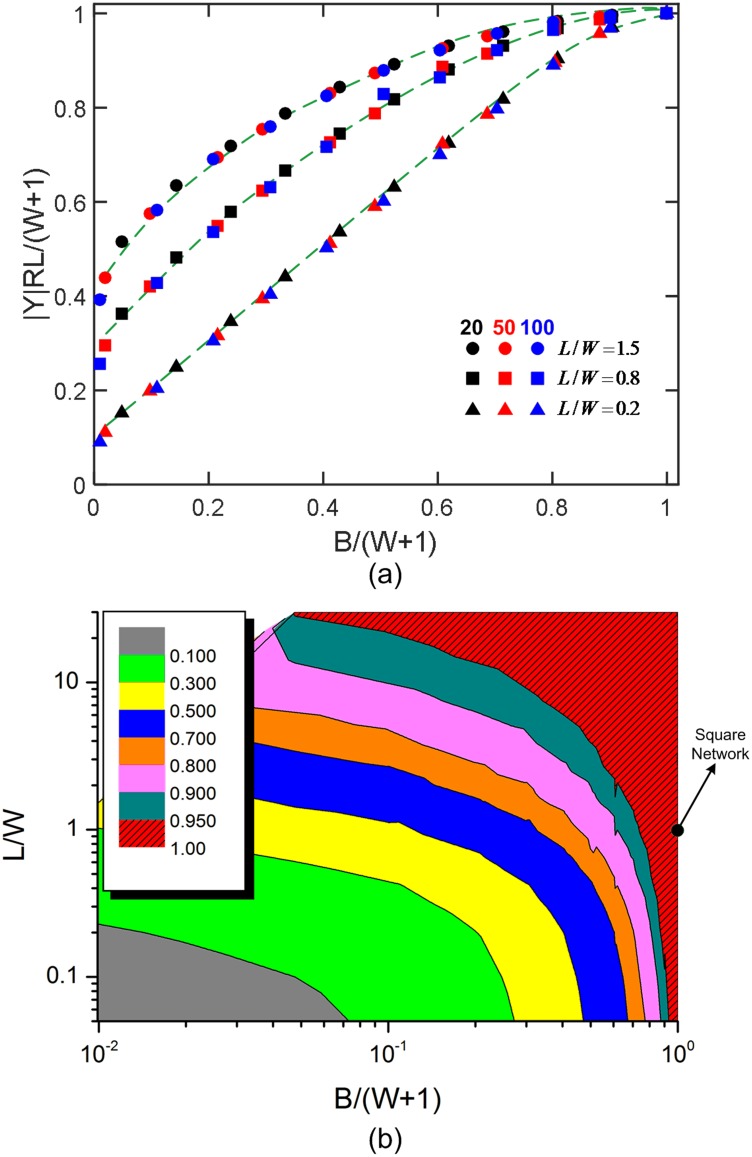
Influences of network aspect ratio and electrode dimension on the values of characteristic admittance. (A) The dependence of characteristic admittance on the *B*/(*W* + 1) ratio, for cases of different widths, *W*, of 20, 50, and 100 but same *L*/*W*, including 0.2, 0.8, and 1.5 (corresponding to ▲, ■, and ●, respectively). (B) Mapping of characteristic admittance values obtained from different-sized networks with varying *L*/*W* and *B*/(*W* + 1) ratios. The square network is marked by the black dot.

We consider 2D rectangular networks with various *L/W* and *B*/(*W* + 1) ratios, in order to study the effects of network size and electrode dimension on frequency dependent responses. The variations of normalized characteristic admittance for rectangular networks (not shown) are comparable to those of square networks. Here, the convergence-divergence behavior is tested with three groups of rectangular networks, which have the fixed width, *W*, of 20, 50, or 100 elements, respectively. The results obtained from the three groups coincide with each other, and typical results for *L/W* = 1.5, 0.8, 0.2 are shown in the Fig 4A. The contour of normalized characteristic admittance shown in Fig 4B presents a clear trend approaching 1, as the network length and the electrode dimension increase. For an RC network with a given size, a smaller value of electrode dimension tends to constrict the current to fewer paths at zones near the electrodes, thus, effectively reducing the network length. However, this influence will diminish as the network lengthens. Networks with large enough length tend to share the same intersection point uninfluenced by the boundary condition. In this case, these networks can be defined as homogeneous systems, with the responses in emergent region unaffected by the network configuration and the electrode dimension. A smaller length/width ratio or electrode dimension will usually lead to normalized characteristic admittance values smaller than 1.

For cases of electrodes of various geometries on the boundary or embedded in RC networks, the influence of the electrodes is mainly induced by the elements connected directly to the electrodes. As the network size increases, boundary elements and electrode-affected elements will have a decreasing percentage. Therefore, the electrode size effect along with the boundary effect will be unobservable for a sufficiently large network. The trend can be found in Fig 4B that the characteristic admittance values for an increasingly large network locate well upwards and to the right from the red zone, asymptotically approaching 1. This universal characteristic can be also extended for an infinite network, with the normalized intersection value to be 1.

The power law dependence of the electrical responses on frequency is of a universal nature for a wide range of complex RC networks. To further interpret this emergent behavior, we investigate the spans of the power-law emergent region. The convergence point tends to be the geometric center of all the emergent regions for various *p*_*c*_. By considering the region center reported here combined with the span, *S*, the emergent scaling behavior region can be well described.

**Fig 5:**
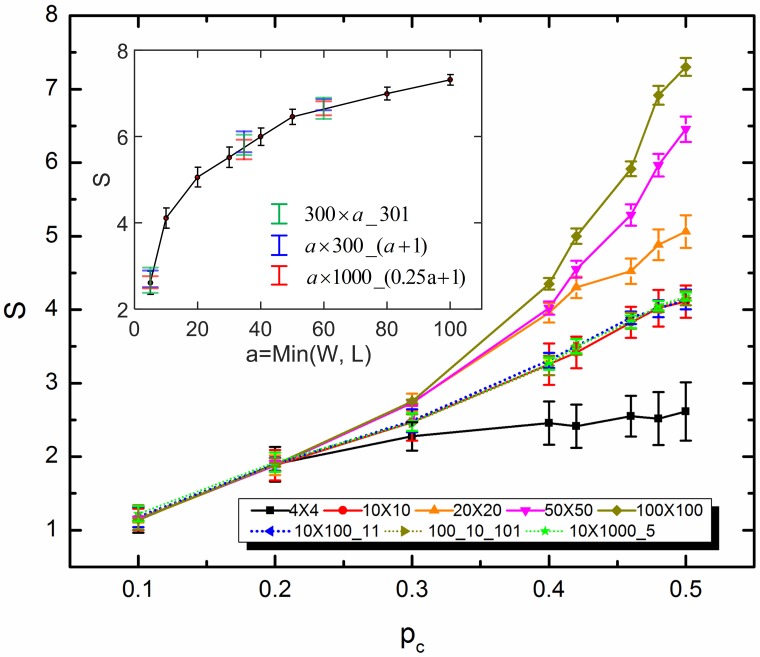
Dependence of the emergent region span on network size and electrode dimension. The results for square networks are shown by solid lines with error bars obtained across ten simulations for each point. Results of rectangular networks (10 × 100_11, 100 ×10_101 and 10 ×1000_5) are presented by dashed lines. The inset compares the spans of different-sized square networks with *p*_*c*_ = 0.5 with those of rectangular networks with various network configurations.

It has been observed [21] that, for a square network with *p*_*c*_ = 0.5, the span of the power-law emergent region increases without bound as the network size increases. In this paper, using the results from square networks as references, we compare the spans of emergent regions obtained for various sized rectangular networks with different electrode dimensions, as *p*_*c*_ varies. We found that when the normalized characteristic admittance value is sufficiently close to 1, it is the smaller dimension, *S*_*min*_ = min(*W*, *L*) that determines the span of emergent region, while the electrode dimension has little influence on the length. Different sized homogeneous networks with the same *s*_*min*_ tend to effectively present identical responses for the emergent region with a given *p*_*c*_. However, discrepancies can be found for responses at low and high frequencies dominated by percolation behavior. This likely to be the case also for an intersection value far away from 1, but with lower accuracy due to the instability of the responses on account of the boundary effects. Only the results with *p*_*c*_ from 0 to 0.5 are discussed and presented in Fig 5, with the consideration of symmetricity of network responses.

The results shown here for varied network and electrode dimensions shed light on the behavior of infinitely-large binary percolation-type network and large networks with irregular boundaries (e.g., in the shape of spline curves) and electrodes (e.g., with the geometry of circular zones, embedded in the network, or unequal-sized electrodes). As long as the network size is significantly larger than the electrode and boundary dimension, the presenting universal features will not be influenced by the boundary and electrode conditions. This enables the network responses to reach a robust and reliable status at the emergent region with the span being determined by the effective network size in the order of $D_{e}{}^{2}$ (*D*_*e*_ is the distance between positive electrode and ground, indicating the shortest current path) and *p*_*c*_ (dominating the slope of universal power law). Additionally, evident trend presented in Fig 5 supports that infinitely large emergent scaling regions can be observed for various capacitor proportions.

## Conclusion

We studied the influences of network geometry and electrode dimension on the electrical responses of rectangular RC networks. The universal scaling behavior can be fully characterized using the center and the length of the emergent region, i.e., the convergence point, and the span, respectively. For both square and rectangular networks, a convergence point is observed at the characteristic frequency, *ω* = 1/*RC*, which usually appears to be the center of the emergent region. At this characteristic frequency, the normalized admittance value |*Y*|*RL*/(*W* + 1) approaches 1 as the length-to-width ratio and electrode dimension increase. For a defined homogeneous network, the span of the emergent range is primarily determined by the shorter dimension of width and length. These observations provide a unified description for the emergent scaling properties of network responses for random two-phase systems with varying topological configurations. The comprehensive understanding of this emergent scaling can guide the design and testing of disordered systems in terms of determining testing conditions (e.g., the shape, size, location, and spacing of the fixtures), boundary conditions, and system dimensions.
